# Supplementary material for: The Influence of Seed Characteristics on Seed Dispersal Early Stages by Tibetan Macaques
Source: Animals (Basel). 2022 May 31;12(11):1416. doi: 10.3390/ani12111416 (PMC9179551; doi:10.3390/ani12111416)
Supplement: Supplementary file 1 [file animals-12-01416-s001.zip › animals-1650024-supplementary.pdf]

Table S1. The physical characteristics of the selected seeds and their fate after ingestion by Tibetan macaques.

Species	Physical characteristics of seeds									Seed fate		
	Size(mm, mean±SD)				Dry Weight (mg, mean±SD)		Volume [b] (mm^3)	Specific gravity [c] (mg・mm^{-3})	Shell weight ratio [d] (mg・mg^{-1})	Total Ingested	Total Recovery	Damage rate (mean ±SD)
	Length	Width	Height	MCD [a]	Weight of seed	Weight of shell						
Litsea pungens	6.42±0.50	6.03±0.60	6.14±0.50	6.19±0.50	129.61±41.04	71.42±24.13	127.77	1.01	0.56	830	2	99.48±0.78%
Syzygium buxifolium	5.94±0.38	5.07±0.46	5.30±0.41	5.42±0.37	60.96±14.06	19.50±3.05	88.81	0.69	0.33	670	0	100.00±0.00%
Lindera glauca	4.88±0.37	4.37±0.29	4.50±0.31	4.58±0.28	40.45±9.91	28.95±5.89	52.60	0.77	0.73	1040	2	99.83±0.33%
Akebia trifoliata	7.59±0.80	4.76±0.48	6.98±0.30	4.45±0.18	59.50±6.05	20.50±3.95	152.40	0.39	0.54	1250	96	92.32±3.48%
Kadsura longipedunculata	4.52±0.39	3.75±0.37	2.66±0.27	3.56±0.31	24.64±6.14	8.51±2.30	27.90	0.88	0.35	1200	82	93.44±1.15%
Rosa laevigata	5.76±0.50	3.15±0.44	2.48±0.41	3.55±0.37	29.69±8.30	25.26±6.94	30.06	0.99	0.85	1240	154	87.60±4.85%
Viburnum dilatatum	3.79±0.26	3.38±0.22	1.63±0.18	2.75±0.18	12.11±2.61	6.11±1.33	14.60	0.83	0.51	1250	452	63.84±3.96%
Celastrus orbiculatus	4.32±0.37	1.95±0.23	1.71±0.18	2.42±0.16	10.28±1.76	7.41±1.25	9.73	1.06	0.72	1150	82	65.63±3.90%
Actinidia chinensis	2.16±0.28	1.35±0.15	0.90±0.10	1.38±0.10	1.61±0.32	0.76±0.15	1.78	0.91	0.48	1500	1036	30.93±7.73%

The physical characteristics were measured on 30 randomly selected seed of each species.

The number of seed feeding repetitions was five (n=5).

[a] MCD was calculated in this formula: $MCD = (a1 \times a2 \times a3)^{1/3}$.

[b] The formula of volume is: $V = \pi R^2(L-2R/3)$, $R = (\text{seed width} + \text{height})/4$, L = seed length.

[c] Calculated as dry weight / volume.

[d] Calculated as shell weight / dry seed weight.
